# Survival and years of life lost in various aetiologies of dementia, mild cognitive impairment (MCI) and subjective cognitive decline (SCD) in Norway

**DOI:** 10.1371/journal.pone.0204436

**Published:** 2018-09-21

**Authors:** Bjørn Heine Strand, Anne-Brita Knapskog, Karin Persson, Trine Holt Edwin, Rachel Amland, Marit Mjørud, Espen Bjertness, Knut Engedal, Geir Selbæk

**Affiliations:** 1 Norwegian National Advisory Unit on Aging and Health, Vestfold Hospital Trust, Tønsberg, Norway; 2 Department of Geriatric Medicine, Oslo University Hospital, Oslo, Norway; 3 Norwegian Institute of Public Health, Oslo, Norway; 4 Faculty of Medicine, University of Oslo, Oslo, Norway; 5 Centre for Old Age Psychiatric Research, Innlandet Hostpial Trust, Ottestad, Norway; Nathan S Kline Institute, UNITED STATES

## Abstract

**Introduction:**

Alzheimer’s disease patients are reported to have higher survival rate compared to patients with vascular dementia or dementia with Lewy bodies. There is a paucity of studies investigating survival including persons with cognitive decline and dementia of various aetiologies.

**Objectives:**

We aimed to compare survival for patients with subjective cognitive decline, mild cognitive impairment, Alzheimer’s disease, vascular dementia, mixed Alzheimer’s/vascular dementia, dementia with Lewy bodies/Parkinson’s disease, and other dementias compared to the general Norwegian population, taking into account the role of gender, cognitive function, function in everyday activities, comorbidity and education.

**Methods:**

Patients (N = 4682), ≥65 years, in the The Norwegian register of persons assessed for cognitive symptoms (NorCog) during 2009–2017 were followed for mortality in the National Registry until January 2018. Flexible parametric survival models were applied to estimate relative survival, life expectancy and years of life lost for diagnostic groups compared with the general population.

**Results:**

Patients with vascular dementia or dementia with Lewy bodies/Parkinson’s had the shortest survival, followed by mixed dementia, Alzheimer’s disease, unspecified dementia, mild cognitive impairment and subjective cognitive decline. At age 70 years, men with vascular dementia or dementia with Lewy bodies/Parkinson’s had life expectancy of 4.7 years, which corresponded to 10.3 years of life lost compared to the general population. Years of life lost for other diagnoses were 10.0 years for mixed dementia, 9.2 years for Alzheimer’s disease, 9.3 years for other dementias, 5.2 years for mild cognitive impairment and 2.2 years for subjective cognitive decline. Corresponding years of life lost in women were: 12.7 years, 10.5 years, 9.8 years, 10.6 years, 7.8 years, and 2.6 years. Poor relative survival among dementia patients was associated with male gender, comorbidity, low cognitive function, and low function in activities of daily living.

**Conclusions:**

Compared with the general population, patients with subjective cognitive decline had no significant loss in life expectancy, while patients with mild cognitive impairment and all dementia subtypes had large losses, especially those with a diagnosis of vascular dementia or dementia with Lewy bodies/Parkinson’s.

## Introduction

Dementia is caused by a variety of brain disorders, which substantially shortens the life span [1], and we have been witnessing a steep increase in deaths due to dementia [2]. Median survival time after a dementia diagnosis varies largely between studies (from 3.2 to 6.6 years) [1], and differ by aetiological dementia type [3, 4]. Knowledge of survival times could be informative for patients, their relatives, and health personnel, but in previous published studies they are often reported as unadjusted estimates, which hampers comparison across studies and dementia types. Furthermore, studies are typically restricted to the most prevalent dementia types such as Alzheimer’s disease (AD) and vascular dementia (VaD), while dementia with Lewy bodies (DLB) and Parkinson’s disease dementia (PDD) is less investigated [3–5]. An exception is a recent study, using data from the Swedish Dementia Registry (SweDem). They found that survival differed across dementia types, with AD having the longest survival [3], which is in line with previous reports [6, 7].

Unfortunately, the Swedish study lacked inclusion of patients with milder cognitive impairment, not fulfilling dementia criteria. AD is usually diagnosed at the dementia stage, but as AD is a progressive disease it can present both at the mild cognitive impairment (MCI) stage and at the even earlier preclinical subjective cognitive decline (SCD) stage [8]. A review from 2006 concluded that there was increased mortality in individuals with a diagnosis of MCI, even if some results were non-significant [9]. More recent studies are also in support of increased mortality for MCI patients [10]. For SCD, the results are particularly scarce and inconclusive [11–13]. In sum, comparison of aetiologies across studies is difficult because it is hard to disentangle if the differences are due to differences in study design, or due to true survival differences between aetiologies.

Several studies report a male survival disadvantage for people with a dementia diagnosis [3, 4, 14], but most of these studies do not correct for the gender differences in non-dementia mortality, and therefore one cannot elucidate if the male survival disadvantage among dementia patients is not merely a reflection of the general male survival disadvantage, or if it is a male disadvantage specific to dementia. To come around this issue, and estimate the “net survival disadvantage” associated with the diagnosis in question, a common procedure is to estimate survival, relative to the general population [15].

In the current study, we aimed to quantify survival for the included dementia types, as well for incident SCD and MCI, expressed as relative survival, life expectancy and years of life lost compared to the general Norwegian population. We also aimed to investigate the role of gender, cognitive function, function in everyday activities, comorbidity and education in relation to survival.

## Materials and methods

### Study population

Clinical data on 4,682 persons ≥65 years from the Norwegian Register of Persons Assessed for Cognitive Symptoms (NorCog) [16], in the period 2009–2017, were linked with the National Registry, and patients were followed from time of diagnosis until death, emigration, or January 1^st^ 2018, whichever occurred first. Those emigrating were censored at date of emigration (n = 5). Apart from this, none of the participants were censored. Patients were followed for maximum seven years.

NorCog is a national register and includes clinical data for patients referred for a dementia workup in outpatient clinics [16]. At the time of linkage, data in NorCog were available from 31 outpatient clinics located across Norway. The register is consent based and has 90% acceptance rate for inclusion [17]. In NorCog the patients undergo several tests of cognition and physical function, their blood is analysed, and a MRI/CT of the brain is performed [18]. If this standard assessment is not sufficient, the patients undergo further investigations to establish a diagnosis. Diagnoses are based on the ICD-10 criteria for research [18]. The Winblad criteria are used to diagnose MCI [19], and patients without cognitive impairment as judged by the cognitive examination receive the diagnosis SCD, in line with SCD criteria [20]. The following cognitive tests are included in NorCog (see reference list in Persson et al [18]): Mini-Mental State Examination (MMSE, screening test for several cognitive domains; global cognitive function); the Clock Drawing Test (CDT, screening of visuospatial function, attention, visual memory, and executive function); the Trail Making Test (TMT) A and B (psychomotor speed and executive function); the Consortium to Establish a Registry for Alzheimer’s Disease (CERAD) 10-word test (immediate and delayed recall and recognition); figure copying from the CERAD constructional praxis exercise (visuospatial skills and delayed visual recall); Similarities from Cognistat (abstraction); the animal-naming test (semantic fluency); the Controlled Oral Word Association Test (COWAT-FAS test; phonemic fluency); and the 15-word short version of the Boston Naming Test (BNT, word retrieval). In addition, the Clinical Dementia Rating scale (CDR), providing a global measure of cognitive and functional impairment is included [18].

### Diagnoses

The study population comprised subjects with one of the following diagnoses: SCD, MCI, AD, VaD, mixed AD/VaD, DLB, PDD, unspecified and other dementias (hereby denoted other dementias). DLB and PDD was treated as one group due to high degree of clinical overlap [21]. In some analyses, all dementia aetiologies were collapsed and denoted dementia. For life expectancy and years of life lost calculations, VaD, DLB and PBB were collapsed due to small numbers for such calculations, and for similar mortality pattern for these diagnosis groups. SCD patients with MMSE score below 25 were regarded as diagnostic error and removed from the analyses (n = 61).

### Covariates

Years of education was dichotomised (0–12, ≥13). Performance in instrumental activities of daily living (IADL) was assessed with the scale published by Lawton and Brody [22], with a summary score of 0–5 for men and 0–8 for women, where low score indicates low function and a high score indicates high function. In the regression models, the IADL-scores were transformed to a 0–100 scale for both genders, with 100 indicating best function, by dividing scores by five for men and eight for women and then multiplying with 100. MMSE [23] was analysed on a continuous scale in adjusted analyses, and grouped in three (0–19, 20–24, 25–30) for presentation. Comorbidity was estimated as a sum of the five disease groups: cerebrovascular disease, cardiovascular disease, cancer, diabetes, chronic obstructive pulmonary disease (COPD).

### National reference population

Mortality rates were compared to those in the general Norwegian population using yearly national life tables from Statistics Norway based on the middle population and number of deaths by gender and one-year ages for each year 2009–2016. For 2017, the life table for 2016 was used. Mortality rates were smoothed using multivariable regression spline models.

### Statistical analyses

Unadjusted survival curves by diagnosis and gender were estimated using the Kaplan-Meyer method. Furthermore, Flexible parametric survival models were applied [24, 25] to estimate overall survival for our patient groups, and survival for the patient groups relative to the general Norwegian population. The flexible parametric models, allow estimation of hazard rates and adjusted survival curves, like the Cox model. Additionally, they allow estimation of relative survival and excess mortality. Excess mortality for dementia patients is the difference between the total mortality of the patients and the mortality that would be expected in the absence of dementia, and could be thought of as the estimated mortality due to dementia after adjusting for mortality due to other causes [15]. Relative survival is the corresponding measure on the relative scale, and is a measure of the survival after a specified period of time, such as 5 years, of the patient groups relative to the survival of the general population. As an example, if 50% of the patient group of interest is still alive after 5 years, while 80% of the general population is alive, then the relative survival is 0.50/0.80 = 0.63. Thus, the survival is 63% in the patient group compared to the survival in the general population.

When comparing survival of several diagnose groups, (let’s say diagnoses A and B) it is feasible to calculate excess mortality rate ratios (RER), which is the absolute excess mortality rate for diagnose A divided by the absolute excess mortality rate for diagnose B. These measures of excess mortality and relative survival are widely used in cancer epidemiology, but should be applicable to other disease groups as well [26].

Another advantage of the flexible parametric model is the possibility to estimate life expectancy and years of life lost for the patient group of interest [27]. Years of life lost was calculated as the difference in life expectancy between the general population and the different patient groups, and might be an intuitive and useful metric for communicating survival statistics, in addition to traditional relative risk estimates.

The flexible parametric models were modelled with five knots for the baseline hazard, with default knot location, and a base model was fitted using diagnosis group as a categorical variable adjusted by age at diagnosis, gender and the interaction diagnosis by gender. Age at diagnosis was first modelled as a restricted cubic spline with three degrees of freedom, but treating age linearly provided equally good fit, so this simpler model was preferred. Further adjustment was done sequentially by adding the covariates to the model. Regression analyses were performed on the study population with non-missing values for all covariates. A similar regression model was applied for the estimation of relative survival, life expectancy, and years of life lost, using smoothed mortality rates from the general Norwegian population as the reference population. In the estimation of life expectancy and years of life lost, year of diagnosis was included in the model as a restricted cubic spline with three degrees of freedom, but also in this setting the simpler linear term had equally good fit, and therefore chosen. Since there were no signs of violation of proportionality of hazards, and models with such effects gave similar results, no time-dependent effects were included in the models. All analyses were performed in Stata version 14.0 (Statacorp, College Station, TX, USA). The project is approved by the Regional Committees for Medical and Health Research Ethics (2015/1510/REK vest) and by the registry owners (NorCog, Norwegian Institute of Public Health and The Norwegian Tax Administration). Written informed consents were obtained from patients.

## Results

### Key characteristics of study sample

The mean follow-up time for the study participants was 3.5 years (median 3.2 years). Total follow-up time was 16,601 person years. Mean age at baseline was 77.1 years (SD 6.7), min age was 65 years and max age was 97 years. Mean age at exit was 80.7 years (SD 6.7) (min 65.2, max 100.5 years).

The most prevalent diagnoses were MCI (35%) and AD (30%), followed by mixed AD/VaD (9%), other dementia (9%), SCD (9%), VaD (6%) and DLB/PDD (4%) (Table 1). The AD and VaD groups had similar mean age (78.0 and 78.4 years), but the VaD group included less women and had a higher crude mortality rate (171 deaths per 1000 py compared to 97 in the AD group) (Table 1). The mixed AD/VaD group was older than the AD and VaD groups (79.6 years), and had crude mortality rate in between AD and VaD. The DLB/PDD group was younger (75.6 years), included more men, and had a crude mortality rate of 144. In comparison, the SCD and MCI groups had mortality rates of 35 and 66, respectively. IADL- function was best in SCD and MCI groups, and poorest among VaD patients. The patients in the VaD group had lowest education, and more comorbidity than the other groups. The MMSE score was lowest in the AD group and highest in the SCD group.

**Table 1:** Background table, N = 4,682 (1,475 deaths).

Diagnosis	Number of patients	Number died	Person years at risk	Deaths per 1000 person years	Mean age at diagnosis (SD)	Women (%)	IADL men (0–5)*	IADL women (0–8)*	Education 13+ yrs (%)	Two or more diseases (%)**	MMSE-score (SD)
SCD	368	56	1580.0	35	73.8 (6.6)	48.6	4.5	7.1	42	26	28.2 (1.5)
MCI	1630	397	6038.6	66	76.4 (6.6)	50.4	3.9	6.4	33	31	25.4 (3.2)
Dementia	2684	1022	8983.3	114	78.0 (6.6)	57.5	2.9	4.9	26	29	21.0 (4.3)
AD	1400	466	4806.9	97	78.0 (6.7)	64.4	3.1	5.1	27	20	20.6 (4.3)
VaD	265	123	717.4	171	78.4 (6.1)	45.3	2.6	4.0	21	60	21.3 (4.1)
Mixed AD/VaD	427	188	1462.0	129	79.6 (6.2)	57.4	2.8	4.7	22	38	21.0 (4.0)
DLB/PDD	198	91	632.6	144	75.6 (6.6)	45.5	2.7	4.6	29	21	21.6 (4.8)
Other dementias	394	154	1364.3	113	77.0 (6.7)	47.7	2.9	4.9	31	31	21.8 (4.6)

*IADL-scoring: http://catch-on.org/wp-content/uploads/2016/12/Lawton_Activities_Daily_living_Scale.pdf. Higher score indicates higher function. Men had 5 items, and women had 8 items.

**Comorbidity: Cerebrovascular disease, cancer, CVD, diabetes, COPD.

### Survival by diagnosis

**Fig 1:**
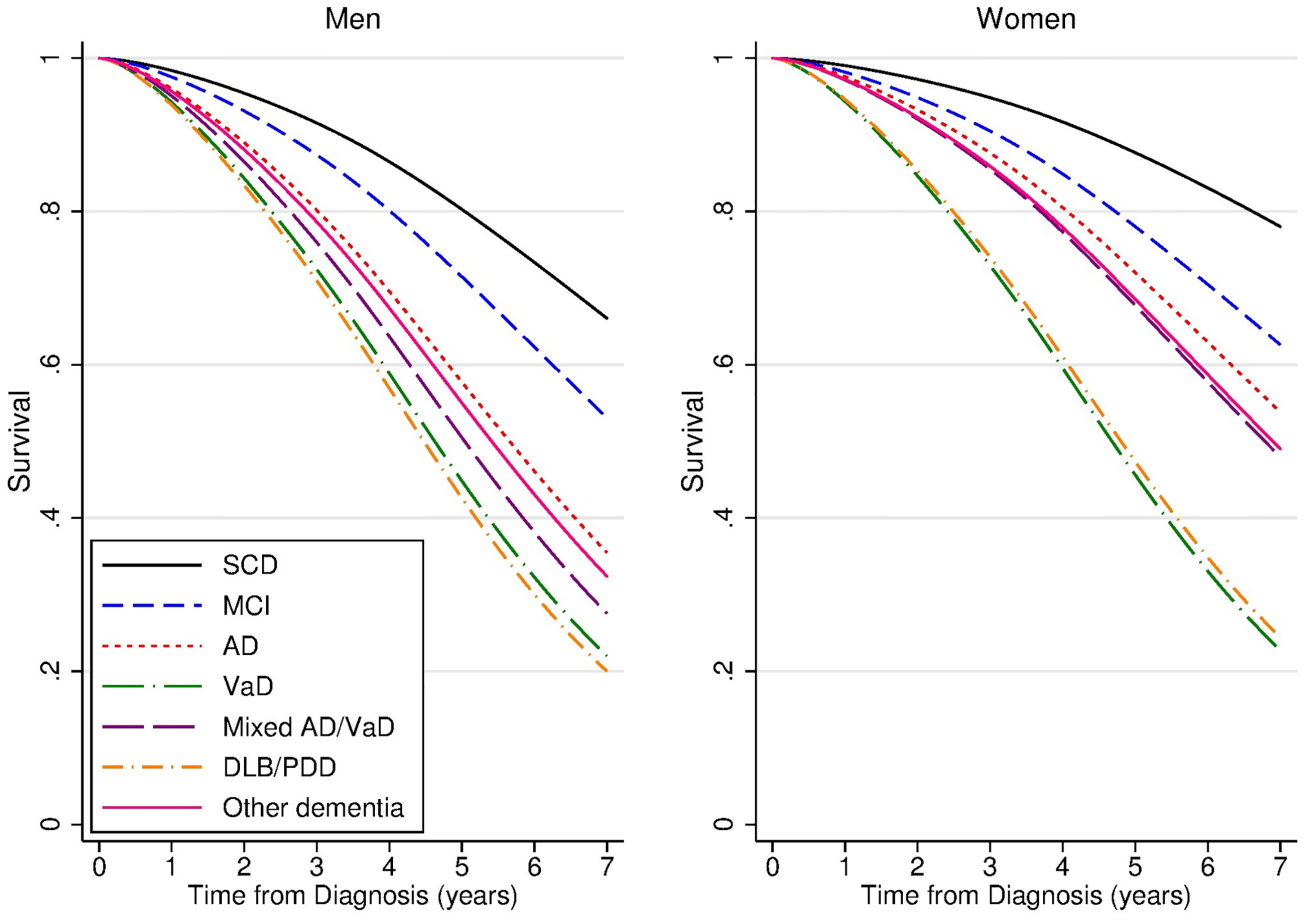
Survival by diagnosis groups at age 75 years, by gender. Modelled using flexible parametric models including diagnosis, age, gender and diagnosis by gender interaction terms. N = 4,682.

Predicted median survival at age 75 years ranged from 4.5 and 4.6 years for male DLB/PDD and VaD patients, respectively, to 12.0 years for female SCD patients (Fig 1, Table 2). Median survival times at age 75 years for dementia patients were 5.2 years (95% confidence interval 5.0, 5.5) in men and 6.8 years (6.4, 7.1) in women (S1 Fig). Corresponding numbers for 80 years old dementia patients were 4.2 years (4.0, 4.5) in men and 5.5 years (5.3, 5.8) in women. Nonparametric Kaplan-Meyer survival curves are presented in the Appendix (S2 Fig).

**Table 2:** Median survival time with 95% confidence interval (CI) at age 75 years by diagnosis and gender. N = 4,682. Modelled using flexible parametric models including diagnosis, age, gender and diagnosis by gender interaction terms.

Diagnosis	Men Median (95% CI)	Women Median (95% CI)
SCD	9.2 (7.5, 10.8)	12.0 (9.1, 15.0)
MCI	7.3 (6.8, 7.9)	8.6 (7.8, 9.4)
Dementia	5.2 (5.0, 5.5)	6.8 (6.4, 7.1)
AD	5.7 (5.2, 6.1)	7.4 (6.9, 8.0)
VaD	4.6 (4.0, 5.2)	4.7 (4.0, 5.4)
Mixed AD/VaD	5.0 (4.5, 5.6)	6.8 (6.0, 7.5)
DLB/PDD	4.5 (3.8, 5.2)	4.8 (4.0, 5.6)
Other dementias	5.4 (4.8, 6.0)	6.9 (6.0, 7.8)

### Survival by diagnosis, relative to the general population: 5-year relative survival (5-yr RS)

**Fig 2:**
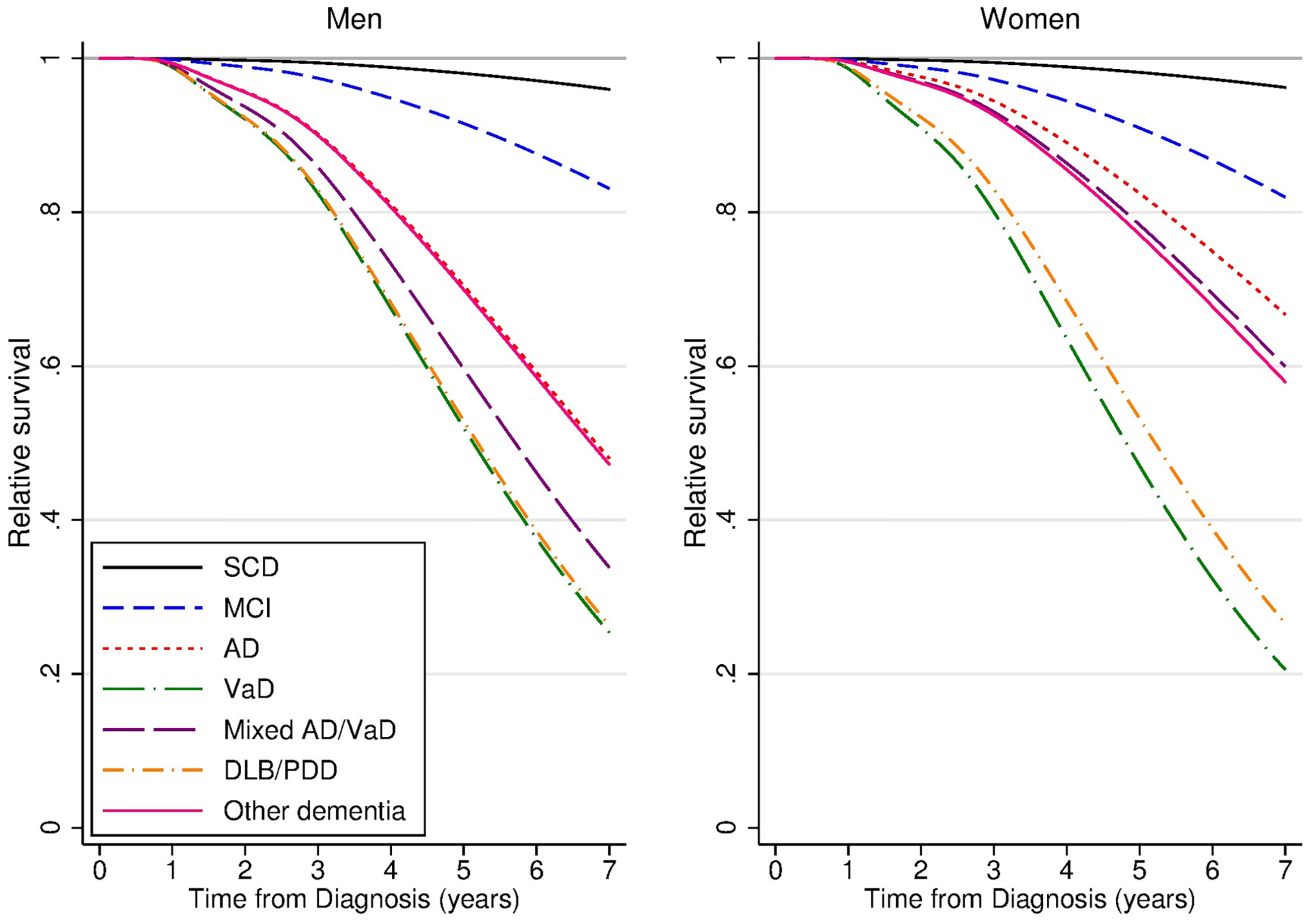
Relative survival of diagnosis groups compared to normal population at age 75 years, by gender. Modelled using flexible parametric models including diagnosis, age, gender and diagnosis by gender interaction terms. N = 4,682.

The survival of the SCD group was not significantly different from the general population (Fig 2, S3 Fig); for men and women, SCD had 5-yr RS of 0.98 (Table 3). Thus, SCD patients had 2% lower five-year survival compared to the survival in the general population. The patients diagnosed with MCI, however, had significantly poorer 5-yr RS; 0.92 (0.86, 0.95) in men and 0.91 (0.87, 0.94) in women. For those diagnosed with dementia the 5-yr RS estimate was 0.66 (0.62, 0.70) for men and 0.77 (0.74, 0.80) for women. For all dementia disorders the 5-yr RS was significantly poorer than the general population. For patients with diagnosis of SCD or MCI we found no gender differences in 5-yr RS, while for patients with dementia, men had significantly poorer 5-yr RS than women, mostly due to poorer survival in AD and mixed AD/VaD.

**Table 3:** Five-year relative survival (5-yr RS) with 95% confidence interval (CI), at age 75 years, for men and women. Modelled using flexible parametric models including diagnosis, age, gender and diagnosis by gender interaction terms. N = 4,682.

Diagnosis	5-yr RS (95% CI) Men	5-yr RS (95% CI) Women
SCD	0.98 (0.71, 1.00)	0.98 (0.79, 1.00)
MCI	0.92 (0.86, 0.95)	0.91 (0.87, 0.94)
Dementia	0.66 (0.62, 0.70)	0.77 (0.74, 0.80)
AD	0.71 (0.63, 0.77)	0.82 (0.78, 0.86)
VaD	0.52 (0.38, 0.64)	0.47 (0.33, 0.60)
Mixed AD/VaD	0.60 (0.48, 0.69)	0.78 (0.69, 0.85)
DLB/PDD	0.53 (0.38, 0.66)	0.53 (0.38, 0.66)
Other dementias	0.70 (0.59, 0.78)	0.77 (0.67, 0.85)

### Excess mortality rate ratios (RER) by diagnosis, and impact of adjustment for covariates

By dividing the excess mortality rates for each diagnosis by the excess mortality for AD, we get estimates for excess mortality rate ratios (RER). Thus, RER for AD was set to 1, and a RER value less than 1 indicates that the excess risk of death for that diagnosis was less than that of AD, and vice versa. In the age and gender adjusted model, with AD as reference group, MCI had RER 0.38 (95% confidence interval (CI) 0.25, 0.56). Thus, the absolute excess mortality rate for MCI was significantly less than that of AD. For VaD the RER was 2.66 (1.91, 3.71) and for DLB/PDD it was 2.46 (1.73, 3.51) (Table 4). Thus, significantly higher than AD. In the fully adjusted model (age, gender, education, comorbidity, IADL and MMSE), the RER for MCI was attenuated and no longer significantly different from AD. Especially the scores on the MMSE and the IADL scales were important for this attenuation; In a model with adjustment for age, gender, IADL and MMSE, the RER for MCI was 0.79 (0.54, 1.17) (not shown in table). RERs for VaD and DLB/PDD, however, were minimally attenuated and still highly significantly higher than AD in the fully adjusted model (Table 4). (Hazard ratios are presented in Supplement, S1 Table).

**Table 4:** Excess mortality rate ratio (RER) with 95% confidence interval (CI) by diagnosis group, step-wise adjusted. Modelled using flexible parametric models.

	RER (95% CI) Full sample, N = 4,682	RER (95% CI) Reduced sample with non-missing values for all co-variates, N = 3,582	
Diagnosis	Age and gender adjusted model	Age and gender adjusted model	Fully adjusted*
SCD	0.07 (0.01, 0.52)	0.11 (0.02, 0.62)	0.35 (0.07, 1.65)
MCI	0.37 (0.26, 0.53)	0.38 (0.25, 0.56)	0.81 (0.55, 1.19)
AD	1.00	1.00	1.00
VaD	2.66 (1.91, 3.71)	2.49 (1.70, 3.65)	1.95 (1.31, 2.91)
Mixed AD/VaD	1.40 (1.02, 1.92)	1.27 (0.89, 1.81)	1.25 (0.89, 1.76)
DLB/PDD	2.46 (1.73, 3.51)	2.32 (1.55, 3.47)	2.03 (1.36, 3.04)
Other dementia	1.20 (0.86, 1.69)	1.20 (0.83, 1.75)	1.30 (0.90, 1.88)

* Adjusted by comorbidity, IADL and MMSE, all on the continuous scale and years of education as a dichotomous variable (0–12, ≥13).

### Life expectancy and years of life lost, by diagnosis

Female life expectancy at age 70 years in the general population in 2016 was 17.3 years, while for women with a dementia diagnosis it was 6.5 years (Table 5). Thus, a significant loss of 10.8 years. Correspondingly, life expectancy at age 70 years in men was 15.0 years in the general population and 5.2 years in men with a dementia diagnosis. Thus, a significant loss of 9.8 years. VaD/DLB/PDD was associated with the lowest life expectancy values in both men and women; 4.6 years in women and 4.7 years in men (Table 5). Mixed AD/VaD was associated with a loss of 10.0 years in men and 10.5 years in women, respectively. AD had life expectancy 7.5 years in women and 5.8 years in men. Thus, significant years of life lost of 9.8 years in female AD patients and 9.2 years in male AD patients. An MCI diagnosis was also associated with significant years of life lost in both men (5.8 years) and women (7.8 years), while SCD was not associated with a significant years of life lost. Also, life expectancy at age 75 years was significantly and substantially reduced in those with dementia and MCI, while SCD did not differ significantly from the general population.

**Table 5:** Life expectancy (LE)* of the general population and by diagnostic group for men and women at age 70 years and 75 years. Years of life lost (YLL) is the difference in LE between the general population and the diagnostic groups (presented with 95% confidence intervals,95% CI). N = 4,689. Modelled using flexible parametric models.

	Men		Women	
Diagnosis	LE*	YLL (95%CI)	LE*	YLL (95%CI)
Age 70 years				
General population	15.0	-	17.3	-
SCD	12.7	2.2 (-2.8, 7.4)	14.7	2.6 (-3.7, 8.9)
MCI	9.2	5.8 (3.8, 8.0)	9.5	7.8 (5.6, 9.9)
Dementia	5.2	9.8 (9.1, 10.6)	6.5	10.8 (9.7, 11.9)
AD	5.8	9.2 (8.2, 10.2)	7.5	9.8 (8.4, 11.3)
Mixed AD/VaD	5.0	10.0 (9.1, 10.9)	6.8	10.5 (9.0, 12.0)
VaD/DLB/PDD	4.7	10.3 (9.6, 11.1)	4.6	12.7 (11.9, 13.5)
Other dementias	5.7	9.3 (8.2, 10.5)	6.7	10.6 (9.1, 12.2)
Age 75 years				
General population	11.5	-	13.4	-
SCD	10.3	1.2 (-1.8, 4.2)	11.9	1.5 (-2.3, 5.4)
MCI	7.8	3.6 (2.3, 5.0)	8.3	5.1 (3.6, 6.6)
Dementia	4.7	6.8 (6.2, 7.4)	5.9	7.6 (6.7, 8.4)
AD	5.2	6.2 (5.4, 7.0)	6.7	6.7 (5.6, 7.8)
Mixed AD/VaD	4.5	6.9 (6.2, 7.7)	6.2	7.3 (6.1, 8.5)
VaD/DLB/PDD	4.2	7.2 (6.6, 7.8)	4.3	9.2 (8.5, 9.8)
Other dementias	5.1	6.4 (5.4, 7.3)	6.0	7.4 (6.2, 8.7)

*LE for year 2016. In this analysis VaD and DLB/PDD was collapsed due to similar mortality pattern and due to small size of these groups.

## Discussion

In this registry based prospective open cohort study, a dementia diagnosis was associated with substantial years of life lost compared with the mortality in the general population, especially for patients with a diagnosis of VaD or DLB/PDD. AD, mixed AD/VaD, other dementias and MCI were also associated with significant loss in life expectancy, while survival for SCD did not differ from that of the general population. Among dementia aetiologies, AD had significantly better survival than VaD and DLB/PDD, also after adjustment for age, gender, education, MMSE, and IADL.

Among the patients who developed dementia after age 75 years, we found median survival times of 5.2 years in males and 6.8 years in females. These survival times are comparable to those reported in a systematic review covering the period 1987–2010 [1]. Furthermore, the mortality rate of 109 deaths per 1000 py in the age group 75–84 in the present study (139 for men and 88 for women) was almost identical to that of 111 per 1000 py (138 for men and 94 for women) reported in SweDem for the period 2008–2011 [3], a period overlapping with our study (in this additional analysis, we limited the follow-up time to the follow-up time in SweDem of 1869 days). Part of this similarity might be because patients in both the SweDem study [3] and NorCog had their diagnosis set at a specialist memory clinic, and that the time-period overlapped. (The SweDem registry also includes patients diagnosed in primary care, but this particular study [3] included only those from memory clinics). Furthermore, the cases in both studies were incident cases. In addition, it should be noted that the general living conditions and the health care systems in Sweden and Norway are comparable.

Our findings of AD having a survival advantage over patients with other dementia diagnoses fits with the conclusion in a review, which included 42 studies, of which 16 compared AD with either VaD or other dementia disorders [4]. The AD survival advantage was also reported in SweDem [3], while a Dutch study (lacking comorbidity, disease severity and cognitive level) did not find such differences [28]. Another similarity between our results and results from SweDem was the ordering of mortality risk for AD, VaD and mixed AD/VaD; AD having lowest risk, VaD having highest, and mixed AD/VaD placed in between. This ordering was also reported in a US study [29]. It has been speculated whether this AD survival advantage over other dementia disorders could be because AD constitutes a “healthier” cohort than other dementias such as VaD and DLB [30, 31]. In SweDem, information on comorbidity was lacking, but adjustment for number of medications as a proxy for comorbidity, and the MMSE score did not explain the AD advantage over VaD and DLB. Similarly, in a recent study by Price et al, the AD survival advantage over DLB persisted after adjustment for age at onset, gender, comorbidity, number of medications, and MMSE [6]. In the present study, only 20% of the AD and DLB/PDD patients had multi-morbidity, compared to 60% in the VaD group. Nevertheless, the AD advantage remained after adjustment for comorbidity and a range of other covariates. Still, we cannot rule out that differences in clinical aspects between the various dementia disorders matter. For example, cerebral infarcts are far more common in VaD, than in AD, as it is a criterion for the ICD-10 VaD diagnosis. Cerebral infarcts increase the overall mortality risk [32]. Furthermore, psychosis is more common in DLB patients than in AD patients, which could increase mortality risk in DLB patients [31]. Nevertheless, adding psychiatric disorders to the comorbidity variable did not alter the findings substantially. Another explanation for the AD advantage could be that AD was diagnosed at an earlier stage than VaD and DLB/PDD. However, controlling for disease severity (CDR) did not change our results (results not shown), suggesting other mechanisms creating this survival disadvantage in DLB/PDD and VaD compared to AD [6]. Lastly, it could be that the neurodegenerative process in AD is slower than in DLB.

Studies on survival of MCI are generally in line with our findings of increased mortality in MCI patients compared with the general population [9, 10]. Our results suggest that the survival advantage of MCI over AD is due to MCI patients’ better cognitive function and IADL-function. One interpretation of this finding is that many patients with a diagnosis of MCI will develop dementia by time [33].

For SCD, the survival did not differ from that of the general population, which was also reported in two previous studies [12, 13], while, a third study reported increased mortality among SCD patients [11]. One explanation for this discrepancy is the age of participants. The study sample in the latter study was substantially older (mean age 81.5 years) than in the present and other studies. People over the age of 80 years have a high risk of developing dementia, and this could explain the higher risk for the SCD patients in the study of Luck et al [11].

Survival after a dementia diagnosis has generally been reported to be in favour of females [3, 4, 14]. However, most previous studies were confined to the cohort of dementia patients, without correcting for the general female survival advantage. Thus, results from many previous studies do not provide information on dementia mortality that is independent of the mortality in the general population. Nor did most previous studies disentangle the effect of possible confounders specifically for dementia, and their impact on all other causes. To come around this, we estimated net survival, which considers survival in the various diagnostic groups compared with survival in the general population [27]. A similar approach was recently applied to investigate relative survival in patients with DLB/parkinson’s dementia in Sweden [5], and their results of 5-year relative survival of 53% was identical to our result. In line with previous reports, the relative measures of survival showed a significantly higher impact of the dementia diagnosis for men compared with women [1, 4]. For specific dementia diagnoses, however, the male disadvantage was limited to AD and mixed AD/VaD. In SweDem, as in our study, there was equality in crude survival across genders for DLB patients [3], while two other studies reported a female disadvantage for DLB [5, 29] and VaD [29]. On the absolute scale, however, dementia was associated with about one year greater loss in remaining life expectancy among women compared with men. This apparent paradox was addressed in a review by Brodaty et al in 2012 [4], and is due to the longer overall life expectancy in women. Thus, women lose more years of their remaining expected life span if they have a dementia diagnosis compared to men.

A limitation of the present study is that patients diagnosed in primary health care were not included, who in general are diagnosed at a later stage of dementia than in a memory clinic. Also patients, who were not competent to give informed consent were not included [34]. Furthermore, routines for referral to a memory clinic in primary care might vary across Norway, resulting in variation in disease severity. However, geographical variation in survival was not addressed in the current study, and we believe the external validity is high for patients competent to give consent, because of the large geographical coverage of the 31 included outpatient clinics. Unfortunately, medicine use was inadequately reported and therefore left out of analyses. However, we did adjust for comorbidity, which would to some extent correlate with number of medications. Strengths of our study are the inclusion of a broad spectrum of reliable cognitive diagnoses made in specialist clinics, as well as inclusion of a control population from the general population. To the latter, as mortality estimates in the general Norwegian population was used to represent mortality for the group without the diagnoses, this will be slightly overestimated as deaths in this group will also include persons with cognitive symptoms or dementia. Another strength is the registry-based design and inclusion of incident cases (newly diagnosed). Since our study is not a population-based survey, but a registry-based study, we minimize the issues of length bias [35]. Participants with rapid cognitive decline will most likely not participate in population-based surveys, and this will result in over-estimation of survival time [35].

All dementia disorders were associated with substantial years of life lost. AD patients had the most favourable survival pattern among those with dementia. MCI was also associated with reduced survival, while SCD did not affect life length significantly. Differences across aetiologies in disease progression and severity, physical and cognitive function and comorbidity were important for the survival differences, but a large part of survival differences between aetiologies remained unexplained.

## Supporting information

S1 Fig Survival by diagnosis groups at age 75 years, by gender. Shaded areas are 95% confidence bands. Modelled using flexible parametric models including diagnosis, age, gender and diagnosis by gender interaction terms. N = 4,682.
(TIF)

S2 Fig Kaplan-Meyer survival curves by diagnosis and gender. N = 4,682.
(TIF)

S3 Fig Relative survival of diagnosis groups compared to normal population at age 75 years, by gender. Shaded areas are 95% confidence bands. Modelled using flexible parametric models including diagnosis, age, gender and diagnosis by gender interaction terms. N = 4,682.
(TIF)

S1 Table Hazard ratios (HR) with 95% confidence interval (CI) by diagnosis group, step-wise adjusted. Modelled using flexible parametric models.
(DOCX)
