# Supplementary figures and images for: Survival and years of life lost in various aetiologies of dementia, mild cognitive impairment (MCI) and subjective cognitive decline (SCD) in Norway
Source: PLoS One. 2018 Sep 21;13(9):e0204436. doi: 10.1371/journal.pone.0204436 (PMC6150521; doi:10.1371/journal.pone.0204436)

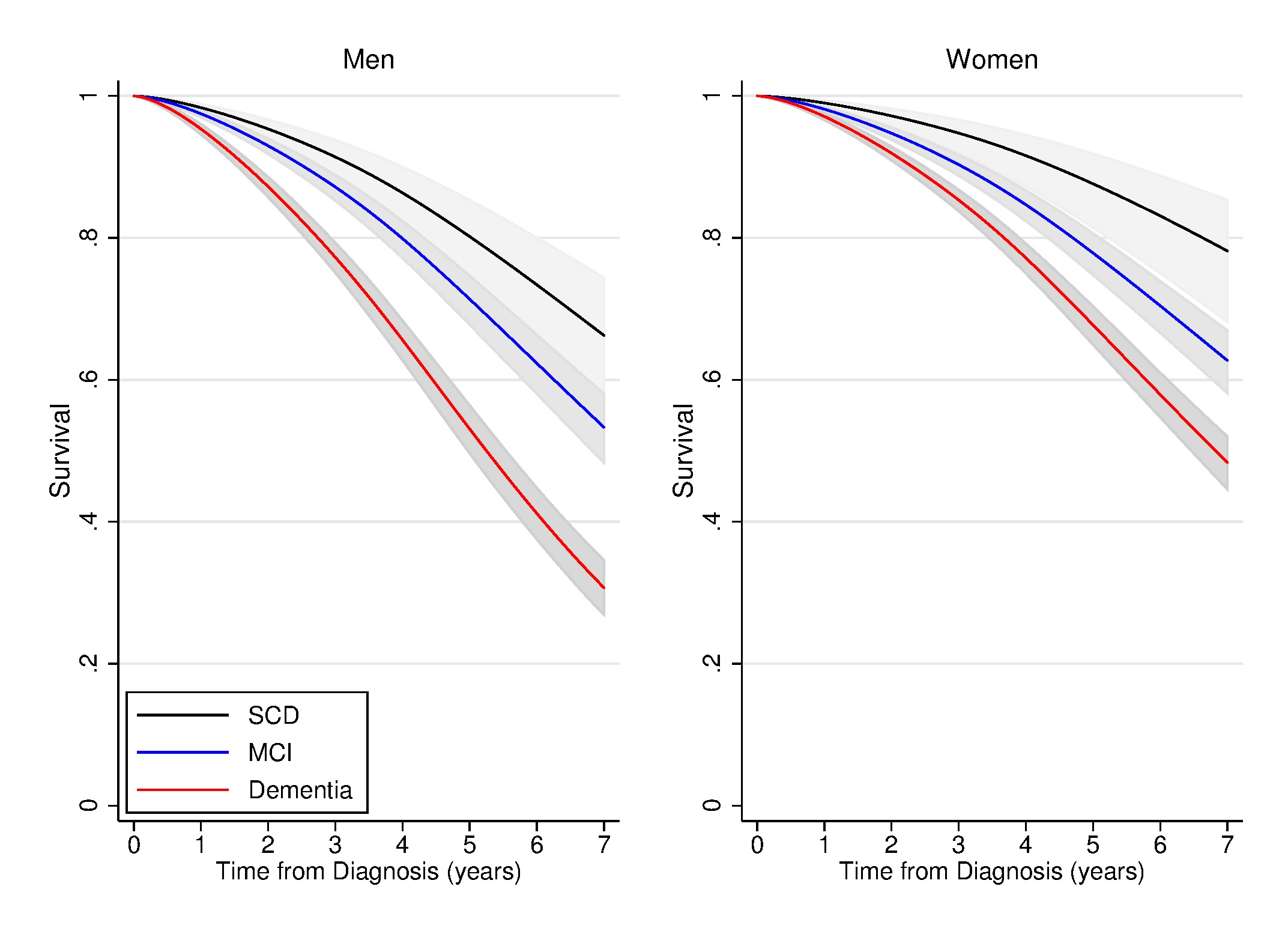

Supplement: S1 Fig — Shaded areas are 95% confidence bands. Modelled using flexible parametric models including diagnosis, age, gender and diagnosis by gender interaction terms. N = 4,682. (TIF) [file pone.0204436.s001.tif]

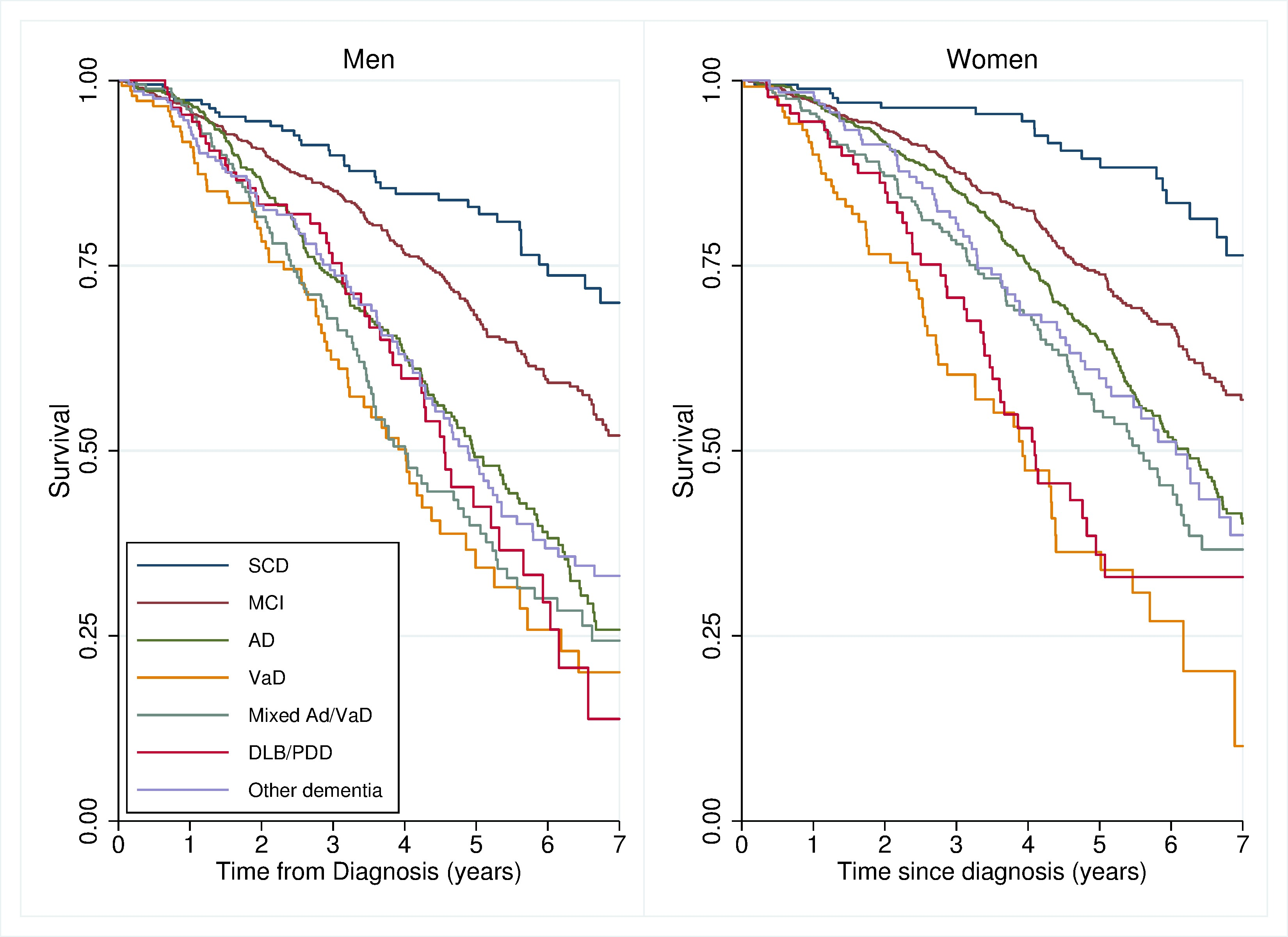

Supplement: S2 Fig — N = 4,682. (TIF) [file pone.0204436.s002.tif]

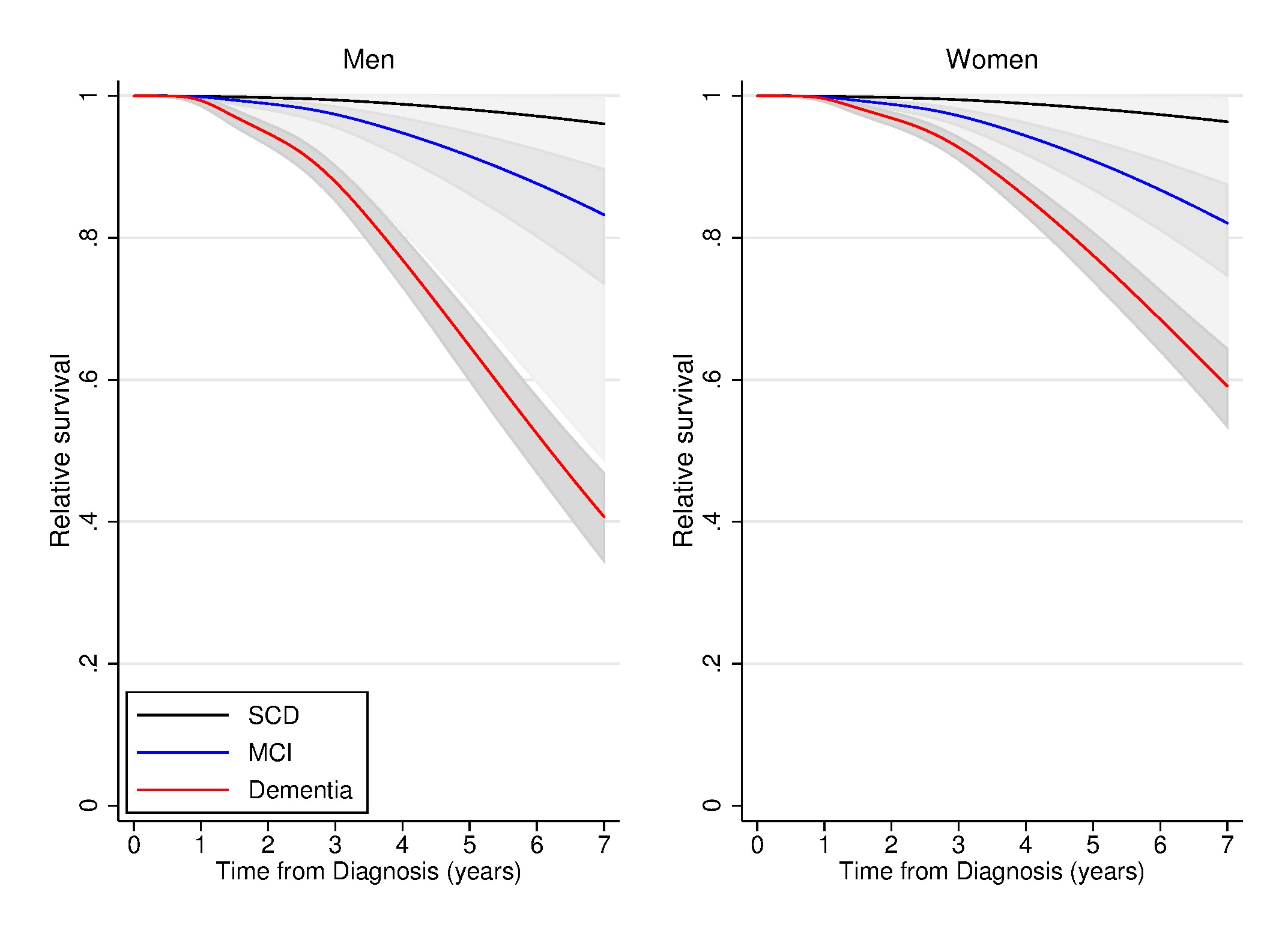

Supplement: S3 Fig — Shaded areas are 95% confidence bands. Modelled using flexible parametric models including diagnosis, age, gender and diagnosis by gender interaction terms. N = 4,682. (TIF) [file pone.0204436.s003.tif]
