# Supplementary material for: Survival and years of life lost in various aetiologies of dementia, mild cognitive impairment (MCI) and subjective cognitive decline (SCD) in Norway
Source: PLoS One. 2018 Sep 21;13(9):e0204436. doi: 10.1371/journal.pone.0204436 (PMC6150521; doi:10.1371/journal.pone.0204436)
Supplement: S1 Table — Modelled using flexible parametric models. (DOCX) [file pone.0204436.s004.docx]

**S1 Table. Hazard ratios (HR) with 95% confidence interval (CI) by diagnosis group, step-wise adjusted. Modelled using flexible parametric models.**

|  | **HR (95% CI)**  **Full sample, N=4,682** |  | **HR (95% CI)**  **Reduced sample with non-missing values for all co-variates,**  **N=3,582** | |
| --- | --- | --- | --- | --- |
| Diagnosis | Age and gender adjusted model |  | Age and gender adjusted model | Fully adjusted* |
| SCD | 0.41 (0.31, 0.55) |  | 0.43 (0.31, 0.61) | 0.77 (0.54, 1.10) |
| MCI | 0.69 (0.60, 0.79) |  | 0.69 (0.59, 0.81) | 0.99 (0.83, 1.17) |
| AD | 1.00 |  | 1.00 | 1.00 |
| VaD | 1.83 (1.50, 2.24) |  | 1.74 (1.37, 2.19) | 1.49 (1.17, 1.90) |
| Mixed AD/VaD | 1.22 (1.03, 1.45) |  | 1.19 (0.98, 1.43) | 1.15 (0.95, 1.39) |
| DLB/PDD | 1.87 (1.49, 2.35) |  | 1.87 (1.45, 2.41) | 1.71 (1.33, 2.21) |
| Other dementia | 1.13 (0.95, 1.37) |  | 1.14 (0.93, 1.41) | 1.19 (0.97, 1.47) |

* Adjusted by comorbidity, IADL and MMSE, all on the continuous scale and years of education as a dichotomous variable (0-12, ≥13).
